# Supporting Self-management Among Young People With Acne Vulgaris Through a Web-Based Behavioral Intervention: Development and Feasibility Randomized Controlled Trial

**DOI:** 10.2196/25918

**Published:** 2021-11-03

**Authors:** Athena Ip, Ingrid Muller, Adam W A Geraghty, Kate Rumsby, Beth Stuart, Paul Little, Miriam Santer

**Affiliations:** 1 Primary Care, Population Sciences and Medical Education University of Southampton Southampton United Kingdom; 2 School of Health Sciences University of Surrey Guildford United Kingdom

**Keywords:** feasibility study, acne vulgaris, intervention study, self-management, primary care, acne, dermatology

## Abstract

**Background:**

Acne is a common skin condition that is most prevalent in young people. It can have a substantial impact on the quality of life, which can be minimized with the appropriate use of topical treatments. Nonadherence to topical treatments for acne is common and often leads to treatment failure.

**Objective:**

The aim of this study is to develop a web-based behavioral intervention to support the self-management of acne and to assess the feasibility of recruitment, retention, and engagement of users with the intervention.

**Methods:**

The intervention was developed iteratively using the LifeGuide software and following the person-based approach for intervention development. The target behavior was *appropriate use of topical treatments*. Barriers and facilitators identified from the qualitative research and evidence from the wider literature were used to identify techniques to improve and promote their use. Young people with acne aged 14-25 years who had received treatment for acne in the past 6 months were invited to participate through mail-out from primary care practices in the South of England in a parallel, unblinded randomized trial. Participants were automatically randomized using a computer-generated algorithm to usual care or to usual care plus access to the web-based intervention. Usage data was collected, and a series of questionnaires, including the primary outcome measure for skin-specific quality of life (Skindex-16), were collected at baseline and at the 4- and 6-week follow-ups.

**Results:**

A total of 1193 participants were invited, and 53 young people with acne were randomized to usual care (27/53, 51%) or usual care plus intervention (26/53, 49%). The response rate for the primary outcome measure (Skindex-16) was 87% at 4 weeks, 6 weeks, and at both time points. The estimate of mean scores between groups (with 95% CI) using linear regression showed a trend in the direction of benefit for the web-based intervention group in the primary outcome measure (Skindex-16) and secondary measures (Patient Health Questionnaire-4 and the Problematic Experiences of Therapy Scale). Intervention usage data showed high uptake of the core module in the usual care plus web-based intervention group, with 88% (23/26) of participants completing the module. Uptake of the optional modules was low, with less than half visiting each (myth-busting quiz: 27%; living with spots or acne: 42%; oral antibiotics: 19%; what are spots or acne: 27%; other treatments: 27%; talking to your general practitioner: 12%).

**Conclusions:**

This study demonstrated the feasibility of delivering a trial of a web-based intervention to support self-management in young people with acne. Additional work is needed before a full definitive trial, including enhancing engagement with the intervention, recruitment, and follow-up rates.

**Trial Registration:**

ISRCTN 78626638; https://tinyurl.com/n4wackrw

## Introduction

Acne is a common condition that is most prevalent among adolescents, affecting >85% of adolescents at some point [1-3]. It can have a substantial physical and psychological impact; however, its main effects are on quality of life (QoL) [4]. First-line treatments for acne are topical treatments that work well at improving acne [5] and have been shown to improve QoL when used appropriately [6,7]. However, studies have highlighted how adherence to topical treatments is poor [8], and discontinuing treatment is associated with a rapid increase in microcomedones, resulting in more acne lesions and subsequent treatment failure [9].

A limited number of interventions have been developed to improve adherence to acne treatments [10-15], many of which have significant shortcomings. A systematic review of the effect of mobile and electronic health technology on adherence [16] (SMS text message reminders [12], telephone call reminders [13], an internet-based education tool [11], and an internet-based survey [14]) found that a weekly internet-based survey was more effective than telephone-based reminders. However, the sample size was small and not powered to determine significance [14]. Other studies included in the review also had small sample sizes ranging between 40 and 61 participants and no power calculations, which may have limited their ability to detect statistically significant differences. To our knowledge, none of these interventions have been informed by theory or developed using robust methods. Interventions developed using theory have proven to be more effective than those without a theoretical base [17].

There is also little information on recruiting through primary care in acne trials. One randomized controlled trial (RCT) investigating the use of supplementary patient educational materials on adherence recruited patients from primary care clinics in the United Kingdom; however, there was no calculation for sample size [10]. As there is very little information regarding uptake and retention rates for this group, further feasibility trials are needed to establish this.

Feasibility trials are an essential part of complex intervention development [18]. However, few interventions for acne have been subjected to feasibility or pilot testing [11,13,19] and, as a result, these trials may have a number of issues around acceptability, delivery, recruitment, and retention and are often small in sample size [20].

In this study, we describe the development of a web-based behavioral intervention to support self-management of acne. We also present the results of a feasibility randomized trial delivering this intervention to young people with acne recruited through primary care.

## Methods

### Development of Web-Based Intervention

The Template for Intervention Description and Replication (TIDieR) guideline [21] was used to facilitate the appropriate reporting of intervention development.

#### Person-Based Approach

The intervention was developed using the Person-Based Approach (PBA) for planning, developing, and evaluating the feasibility of the intervention [22]. The aim of this method is to ground the intervention in the views and experiences of the people who will use it to ensure that it is persuasive, accessible, and engaging for the target population [22]. The PBA involves in-depth qualitative research to identify key objectives and barriers and facilitators to target behaviors [22]. We carried out a systematic review and synthesis of qualitative research to explore the qualitative literature on acne among patients, carers, and health care professionals [23]. The review protocol was registered on PROSPERO (International Prospective Register of Systematic Reviews; registration number CRD42016050525). A secondary analysis of qualitative interviews with people with acne was also carried out to understand young people’s views and experiences with acne and its treatments [24].

#### Creating Guiding Principles

Alongside intervention planning, guiding principles were drafted and iteratively developed throughout, identifying distinctive intervention features to address these. This method involved highlighting key objectives from qualitative research (1). to support young people in gaining autonomy and competence around acne management, (2) to support and promote autonomy in making treatment choices, and (3) to provide support and acknowledge the psychological impact of acne (see Table 1 for guiding principles developed for this intervention).

**Table 1:** Guiding principles.

Key intervention objectives	Patient characteristics	Evidence for key behavioral issues	Guiding principles	
			Design objectives	Key (distinctive) intervention features
To improve the lives of young people with acne To promote self-management of acne To promote the appropriate use of topical treatments	Young people who have mild to moderate acne vulgaris	Little knowledge about acne and its treatments (QR)^a^ Young people can be confused with the myths and misconceptions around acne and are unaware or unwilling to acknowledge that acne requires ongoing treatment. Low motivation to engage with long-term treatment (QR) Certain beliefs about the causation of acne may affect people’s perceived necessity of treatment. Difficulty judging efficacy of topical treatments (QR) Belief that topical treatments do little and are only keeping their acne at bay may result in early abandonment of treatment. Difficulty overcoming barriers (QR) Young people can be uncertain about how to manage side effects of treatment, financial constraints, lengthy routines, and uncertainties around how to use medication. Confusion between cosmetic and medical treatments for acne (QR) Young people perceive they have tried all the topical treatments available	To support young people in gaining autonomy and competence around acne management	Offer users choice wherever possible Minimize disruption to lifestyle Dispel myths and misconceptions about the causes of acne Autonomy-supportive language Ensure they have a complete understanding of acne and the rationale behind their treatment To build their self-efficacy for the target behaviors (eg, 4-week challenge to support patients to formulate a personal goal or action plan, advice on how to minimize side effects including skin irritation, and a video with step-by-step instructions on how and when to apply topical treatments) Educational information or rationale supported by scientific evidence (topical treatments are equally as effective as antibiotics) Stories and testimonials to model successful management using topical therapies Addressing common concerns Provide a list of topical treatments and explain how they work
		Need for control over treatment choice and disease (SR)^b^ Young people want control over their treatment choice as well as their condition and this has been shown to improve adherence and psychological impact	To support and promote autonomy for making treatment choices	Provide advice on how people can effectively communicate with their GP^c^ Invite, acknowledge, and value views or preferences (eg, CAM^d^ therapies) Provide a list of topical treatments and explain how they work Offering user choice wherever possible Autonomy-supportive language throughout
		Difficulty dealing with psychological issues (SR and QR) Young people can be unsure about how to cope with the psychological impact of acne, including depressive symptoms, stress, anxiety, and embarrassment Difficulty presenting psychological issues to HCP^e^ (SR) Young people may be unwilling to present psychological problems to their HCP	To provide support and acknowledge the psychological impact of acne	Acknowledge the psychological impact of acne (eg, (1) emphasize that everyone with a skin disease can be at risk of psychological symptoms and (2) provide patient stories about how they dealt with the impact of acne) Provide advice on how people can effectively communicate with their GP Provide advice on different coping strategies

^a^QR: qualitative research (barriers identified from the secondary analysis of qualitative interview data [24]).

^b^SR: systematic review (barriers emerged from systematic review and synthesis of qualitative papers on acne) [23].

^c^GP: general practitioner.

^d^CAM: complementary and alternative medicine.

^e^HCP: health care practitioner.

#### Target Behavior

The hypothesized outcome of the intervention was to improve QoL for young people with acne through the target behavior *appropriate use of topical treatments*. This target behavior was chosen as it has been shown that effective use of topical treatments can improve acne [5] and benefit QoL [6,7]. For addressing this target behavior, barriers and facilitators identified from the qualitative research were described along with the proposed intervention element.

Evidence from the literature and qualitative research (including the systematic review and synthesis of qualitative data [23] and the secondary analysis of interview data with young people [24]) highlighted several barriers to the appropriate use of topical treatments that needed to be addressed in the intervention. These included concerns about side effects, confusion about the different types of topical treatments, beliefs around the ineffectiveness of topical treatments, belief that acne is a short-term condition that will resolve on its own, confusion about how to use treatment, the time-consuming nature of topical treatments, and the belief that oral treatments were more effective than topical treatments.

#### Behavioral Analysis

Alongside the PBA, a behavioral analysis was carried out to map the intervention components to the behavior change taxonomy, which is a list of consensually agreed techniques for specifying interventions [25]. The behavioral analysis showed that the intervention targeted nine behavior change techniques from the 93 behavior change taxonomies [25]. A central behavior change technique was *instructions on how to perform the behavior* in terms of advice about choosing the right topical treatment and instructions and demonstrations on how to use topical treatments appropriately. The intervention components were also mapped onto the COM-B model, part of the behavior change wheel [26], to map the target constructs and functions for the intervention [26]. This included six target constructs (physical capability, psychological capability, physical opportunity, social opportunity, automatic motivation, and reflective motivation) and five intervention functions (persuasion, education, training, enablement, and modeling).

Qualitative research showed the Extended Common Sense Model of Illness [27] to be a useful model for understanding how people with acne conceptualize illness and treatment and was therefore used in the behavioral analysis to check that all important components of the model were covered in the intervention (Table 2).

**Table 2:** Behavioral analysis of Spotless intervention.

Barrier or facilitator for target behavior^a^ and intervention component		Spotless module	Target construct (BCW)^b^		Intervention function (BCW)		Behavior change technique (using 93 BCTTv1)^c^		Target construct (ECSM)^d^	
**Concerns about side effects from topical treatments (eg, dry skin and bleaching; QR^e^ and SR^f^).** **Fabbrocini et al [28]: having no side effects was reported as one of the most important attributes of topical treatments (EBL^g^)**										
	Provide persuasive and credible information about the side effects of topicals and their safety via scientific evidence and personal stories	Core treatments	Psychological capability, reflective motivation, and social opportunity		Education, persuasion, and modeling		5.1. Information about health consequences 6.2. Social comparison 6.3. Information about others’ approval 9.1. Credible source		Beliefs about necessity and concerns over its use	
	Provide advice on how to choose the right topical	Core treatments	Psychological capability		Training and education		4.1. Instructions on how to perform the behavior		Beliefs about necessity and concerns over its use	
**Confusion about the different types of topical treatments resulting in difficulty with making own treatment choices (QR and SR)**										
	Provide advice on how to choose the right topical	Core treatments	Psychological capability		Training and education		4.1. Instructions on how to perform the behavior		Curability or controllability	
	Provide information about different topicals (eg, most common or least common topicals and how they work)	Core treatments	Psychological capability		Education		5.1. Information about health consequences		Curability or controllability	
**Belief that topical treatments do little to help as they are only *keeping their acne at bay* (QR)**										
	Provide persuasive and credible information about the effectiveness of topicals via scientific evidence and personal stories Provide rationale for how topicals control acne Explain via personal stories or video that it can take time for topical treatments to work	Core treatments	Psychological capability, reflective motivation, and social opportunity		Education, persuasion, and modeling		5.1. Information about health consequences 6.2. Social comparison 6.3. Information about others’ approval 9.1. Credible source		Beliefs about necessity	
	Provide a chart for them to monitor how their skin is after applying topical treatments each day as part of the 4-week challenge	Core treatments	Reflective motivation		Education and persuasion		5.1. Information about health consequences 2.3. Self-monitoring of outcomes of behavior		Beliefs about necessity	
**Belief that acne is a short-term condition caused by puberty and therefore it will go away on its own (QR); McNiven [29]: belief that acne is a cosmetic problem rather than a medical condition (EBL)**										
	Provide information on the causes of acne and dispel misconceptions using a myth-busting quiz Provide persuasive and credible information about how acne can be effectively managed using treatment, including scientific evidence and personal stories Provide information about what acne is, the importance of treating it early, and information about referrals Provide advice on when to see an HCP^h^ about acne Provide advice on speaking with an HCP about acne	Myth-busting quiz; What are spots or acne; Talking to your GP^i^	Psychological capability, reflective motivation, social opportunity, and physical opportunity		Education, modeling, persuasion, and training		4.1. Instructions on how to perform the behavior 5.1. Information about health consequences 6.2. Social comparison 6.3. Information about others’ approval 9.1. Credible source		Cause, timeline, and identity	
**Lack of skills regarding how to apply topicals and for how long (QR); Myhill et al [10]: supplementary patient education materials and video about application of topical treatment led to improved adherence (EBL); Sandoval et al [19]: education via physical demonstration led to 15% overall higher adherence rates (EBL)**										
	Provide written instructions and an instructional video on how to use topical treatments correctly	Core treatments		Physical capability, social opportunity, and reflective motivation		Training, modeling, and persuasion		4.1. Instructions on how to perform the behavior 6.1. Demonstration of the behavior 6.2. Social comparison 6.3. Information about others’ approval 9.1. Credible source		Concerns over its use
	4-week challenge: provide a chart to help people record their skin condition when they have used their topical treatment each day	Core treatments	Reflective motivation		Education and persuasion		5.1. Information about health consequences 2.3. Self-monitoring of outcomes of behavior		Concerns over its use	
**Belief that topicals are time-consuming to apply (QR); Rueda [15]: simplifying regimen and considering patient preference increases adherence (EBL)**										
	Provide information on how to incorporate topicals in everyday life Reassure people that applying topicals should not be time-consuming Advise people to plan when to apply their topical Suggest applying their topical at the same time in the same context each day	Core treatments	Psychological capability and automatic motivation		Education and enablement		1.4. Action planning 4.1. Instructions on how to perform the behavior 5.3. Information about social and environmental consequences		Concerns over its use	
**Belief that tablets are easier, stronger, and quicker to take effect than topicals (QR); Santer et al [30] found that some participants preferred oral treatments as they perceived these to be *stronger* than topicals (EBL)**										
	Provide persuasive and credible information about the effectiveness of topicals and antibiotics via scientific evidence and personal stories Provide information about the consequences of long-term oral antibiotic use	Core treatments and antibiotics	Psychological capability, social opportunity, and reflective motivation		Education, modeling, and persuasion		5.1. Information about health consequences 6.2. Social comparison 6.3. Information about others’ approval 9.1. Credible source		Concerns over its use	

^a^Target behavior: appropriate use of topical treatments.

^b^BCW: behavior change wheel.

^c^BCTTv1: behavior change technique using the Behavior Change Technique Taxonomy (v1).

^d^ECSM: Extended Common-Sense Model of Illness.

^e^QR: qualitative research (barriers identified from the secondary analysis of published interview data; evidence-based literature).

^f^SR: systematic review (barriers emerged from systematic review and synthesis of qualitative papers on acne); qualitative research.

^G^EBL: barriers and facilitators emerged from a review of literature on acne (including studies testing the effectiveness of interventions to improve adherence to acne treatments).

^h^HCP: health care practitioner.

^i^GP: general practitioner.

#### Web-Based Intervention

The web-based intervention, Spotless, was developed using the LifeGuide software [31]. The intervention was delivered on the web via the internet and included a compulsory core module on topical treatment. This included information about the different types of topical treatments available, how they work, how to use them appropriately, common side effects, and how to manage them. Information was adapted from accurate web-based sources, including National Health Service [32], National Institute for Health and Care Excellence [33], and the British National Formulary [34]. This was initially carried out by artificial intelligence, and the team (MS, AG, PL, and IM) provided suggestions throughout. The purpose of adapting the information was to ensure that it was easily understood by young persons. An example of this was using information about types of treatments, including how they are used and the side effects, but rewriting this in lay language. Six optional modules were highlighted as important for the self-management of acne in earlier qualitative studies (Textbox 1).

**Overview of intervention.**

**Overview**

**Figure 1:**
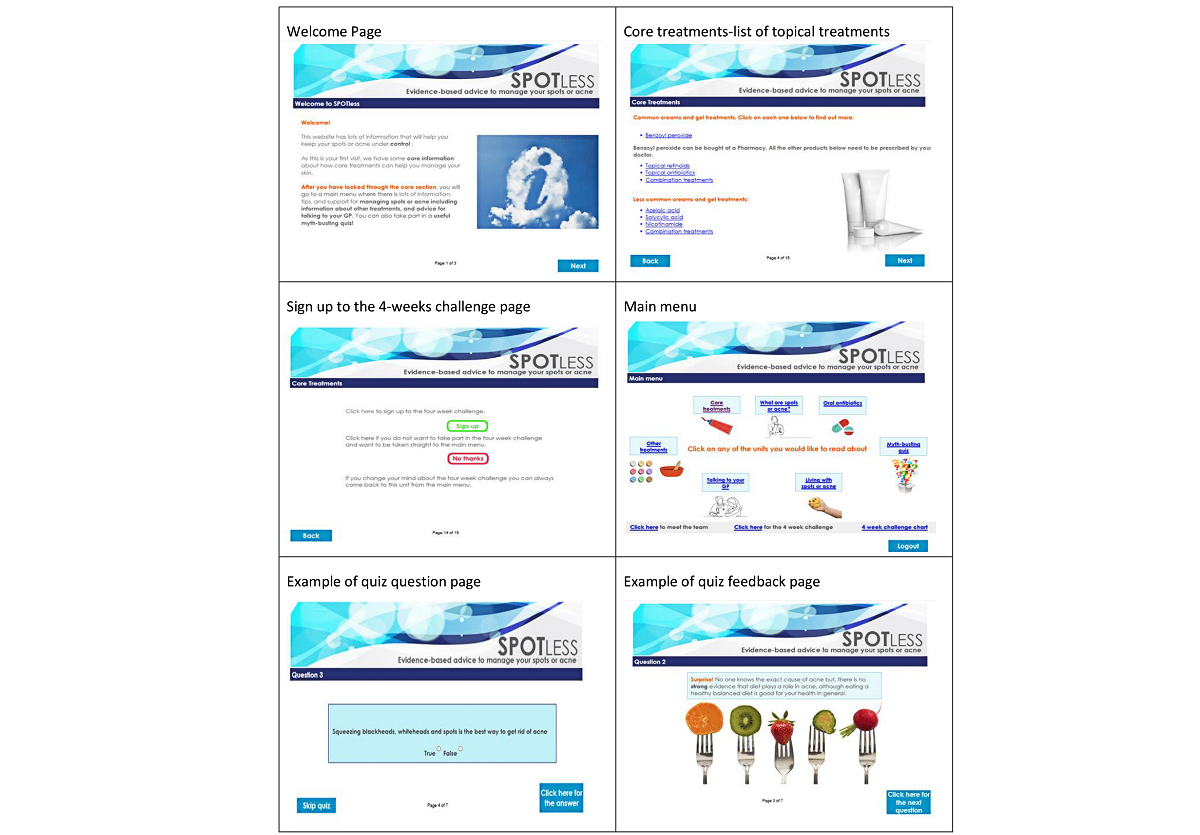
Screenshots of the Spotless website.

- When participants first visit the website, they are taken to a *core module* on topical treatments. In the module, they have the option to take part in a 4-week challenge using their choice of topical along with the advice from the website. After completing this module, participants are taken to a main menu page with six optional modules, which they can visit as many times as they want throughout the course of the study. These include *What are spots or acne, Myth-busting quiz, Oral antibiotics, Living with spots or acne, Talking to your general practitioner, and Other treatments* (see Figure 1 for screenshots of the website).
- After the initial visit, participants are taken directly to the main menu page, where they can choose which modules to explore with the option of looking at the *core module* again.
- The intervention includes a *Meet the team* page where participants are able to see who developed the website (general practitioners, psychologists, and academic researchers); quotes adapted from qualitative research and relevant statistics are presented throughout the intervention, and a downloadable chart is available to help participants self-monitor their progress during the 4-week challenge. The intervention also includes audio, visual, and interactive features including a *myth-busting quiz* where participants can answer questions about popular myths and misconceptions around acne.

#### Intervention Optimization Using Think-Aloud Interviews

As part of the development stage, think-aloud interviews [35] were carried out with 19 participants with acne using the draft intervention to gather feedback and further modify the intervention. Participants were recruited through mail-out from primary care practices, opportunistic sampling using posters, and advertising via social media. The inclusion criteria for the study were young people aged 14-25 years with acne or those who had consulted about their acne or obtained a prescription for their acne in the past year. Potential participants were excluded if they were outside the age range or did not have acne. General practitioners (GPs) were also asked to screen lists to ensure that the invitation pack was not sent to patients where they felt this would be inappropriate. Face-to-face think-aloud interviews were conducted by following a semistructured interview guide to ensure that all topics were covered while also allowing participants to discuss any concerns they had about the intervention. This process involved asking participants to use the intervention while speaking out their thoughts aloud. Interviews were transcribed and analyzed using a deductive approach to code the data using the objectives of the study (engagement, persuasiveness, and usability) and identify positive and negative comments to aid intervention development.

Overall, participants found the intervention engaging, persuasive, and usable, with some suggestions for changes. Main changes made as a result of the interviews were adding pseudonyms and ages to quotes (these quotes were adapted from the qualitative interview study [24] and included to provide other peoples’ experiences in managing acne); changing the context of certain quotes to make them more relatable to the intended user; providing further clarification on how people can manage sun sensitivity as a potential side effect of topical treatments; further clarification on steps for applying topical treatments (time of day and quantity) and what *sensitive areas of the face* referred to; changing the 6-week challenge to 4 weeks as some participants felt that 6 weeks would be too long to commit and based on evidence that topical treatments could take effect sooner [10]; changing the core module name from *universal core treatments* to *core treatments* so that participants would not misinterpret the website as advertising something; and changing the layout of the intervention including the banner, images, and color scheme.

### Patient and Public Involvement

Two public contributors aged 24 and 26 years with experience of acne provided input throughout to enhance the usability and accessibility of the intervention. This included providing feedback to further enhance the intervention before the feasibility trial, commenting on participant facing documents, and advising on the choice of the primary outcome measure for the trial. Comments about the intervention were both positive and negative regarding the layout, content, and appropriateness of the website for the target population. One contributor commented on their preference of the primary outcome measure for the feasibility trial and opted for Skindex-16 [36] over various other skin-specific QoL measures for reasons including the appropriateness of the questions. Input on the participant facing documents led to changes in wording, making it more appropriate for a layperson and for the target population.

### Feasibility Study

#### Trial Design

This was a randomized, unblinded feasibility trial comparing two parallel groups: usual care and usual care plus web-based intervention.

#### Study Population and Eligibility Criteria

The intervention was aimed at young people with acne managed through primary care in the United Kingdom. Participants were recruited through mail-outs from 20 GP practices in the South of England to people aged 14-25 years whose electronic record included a diagnosis of acne and who had received one or more prescriptions for acne in the past 6 months.

People who had previously taken part in the think-aloud study were excluded, as were people who said their acne had cleared and those taking oral isotretinoin, as it is not recommended to use topical acne treatments at the same time as isotretinoin because of the side effects of dry skin.

#### Procedure

Patients aged ≥16 years, who met the criteria, were sent an *adult study pack* from their GP, and patients <16 years received a *child study pack* (addressed to the parent or carer). Initially, the pack included an information sheet, a freepost envelope, and a covering letter. Those interested returned a reply slip, and a member of the study team contacted the participant, providing them with a unique participant identification number and the link to the web-based intervention. Amendments were made to the process, and these were approved by both the university and National Health Service ethics committees. Changes included an additional A5 flyer about the study to appeal to the target population and a sign-up sheet providing participants with their unique identification number and a link to the intervention. These changes were essential for assessing the feasibility of the study with a challenging population to recruit. Implied parental consent was approved for participants aged <16 years as invitation letters were sent to the parents; therefore, passing log-in details to their child implied consent. This is because young people from 14 years usually self-manage their acne and are responsible for using topical treatments themselves. The link directed all participants to further information and a web-based consent procedure. After consenting, participants were asked to complete a set of baseline questionnaires before being randomized into 1 of 2 groups. Follow-up questionnaires for the trial were conducted at 4 and 6 weeks as a recent study suggested that topical treatments could take effect within 1 to 4 weeks and that continuation after the 4 weeks would lead to further improvements [10]. Participants received an automated email followed by a reminder email a week after (5 and 7 weeks) if they had not completed these. Further text and subsequent phone follow-ups were conducted for nonresponders to complete the outcome measures, particularly the primary outcome Skindex-16.

#### Intervention and Comparator

The usual care group received treatment as usual from their GP, including appointments, prescriptions, and referrals to the dermatologist, if necessary. Participants in this group were given access to the intervention after they had completed the 6-week follow-up questionnaires.

Participants in the usual care plus web-based intervention group received care as usual with immediate access to the website as described to help them self-manage their acne.

#### Outcome Measures

We sought to assess a range of feasibility outcomes including the following:

- The rate of recruitment and the number of practices required
- Completion rates of questionnaire outcome measures
- The acceptability of measuring skin-specific QoL using Skindex-16
- The feasibility of a range of quantitative measures
- Intervention usage in terms of number of log-ins and modules accessed

Outcome measures included the following: Skindex-16 [36] was included as a skin-specific QoL measure. Skindex-16 is a validated measure that includes 16 items on a 6-point Likert scale ranging from 0 (never bothered) to 6 (always bothered), which are transformed into a 100-point scale, with higher scores indicating a lower level of QoL [36].

EQ-5D-5L [37] was included as a health-related QoL measure collected at all intervals. It comprised five domains (mobility, self-care, usual activities, pain or discomfort, and anxiety or depression) with five response levels (no problems, slight problems, moderate problems, severe problems, and extreme problems) that describe the current health state. The visual analog scale was also included alongside this [37].

The Problematic Experiences Therapy Scale (PETS) [38] was included and data collected at each interval to explore barriers to treatment adherence. This measure includes 12 items with four subscales: *problems due to symptoms*, *problems due to uncertainty about therapy*, *problems due to doubts about treatment efficacy*, and *practical problems*. Participant responses were scored on a scale ranging from 1 (disagree strongly) to 5 (agree strongly), with higher scores indicating fewer barriers to adherence [38].

Participants also completed the Credibility/Expectancy Questionnaire at baseline as a process predictor. It measures how a person thinks and feels about their therapy and its likely success [39]. These are measured using two types of rating scales, one from 1 (not at all) to 9 (very much) and another from 0% (not at all) to 100% (very much), and it provides an overall score ranging from 3-27 for each factor [40].

The Patient Health Questionnaire-4 (PHQ-4) was used to measure anxiety and depression [41] collected at all intervals. This brief screening tool has been shown to be a reliable and valid measure in young people [42] and includes 4 items measured on a 4-point Likert scale ranging from 0 (not at all) to 4 (nearly every day) [41].

Treatment monitoring questions were included in order to collect data on what topical treatments participants were using, whether they experienced side effects, how they dealt with these, how often they were using treatment, and any other treatments they were using for their acne.

Sociodemographic questions included age, gender, education, age of onset of acne, and whether living with parents or independently.

### Sample Size

The target sample size was 65 participants, with 40 in the intervention group and 25 in the usual care group. This was deemed appropriate as guidance on sample sizes in feasibility trials ranged from 12 to >30 participants in each arm [43,44].

### Randomization

We intended randomizing all participants into 2 groups in a 2:1 ratio using a computer-generated algorithm. However, because of an error in the randomization software, the block randomization was changed to a 1:1 ratio. The sequence was concealed as this was all done via a computer.

### Data Collection and Analysis

Data were automatically collected via the LifeGuide software [31], including information about recruitment, number of log-ins, and which modules or pages participants had accessed. Descriptive statistics were used to describe the data, and outcome measures were analyzed using SPSS version 25 [45]. Linear regression, adjusting for baseline scores, age, gender, education, and age of onset of acne, was performed to provide estimates of mean scores between groups (with 95% CIs). Intention-to-treat analysis was used, including all participants who were randomized, without imputing missing data. There was no significance testing, as this was a feasibility trial and was not sufficiently powered to seek differences between groups.

### Ethics Approval

The feasibility trial was approved by the National Research Ethics Service Committee east of England (ref: 18/EE/0105) and registered on the ISRCTN registry (78626638).

## Results

### Recruitment

**Figure 2:**
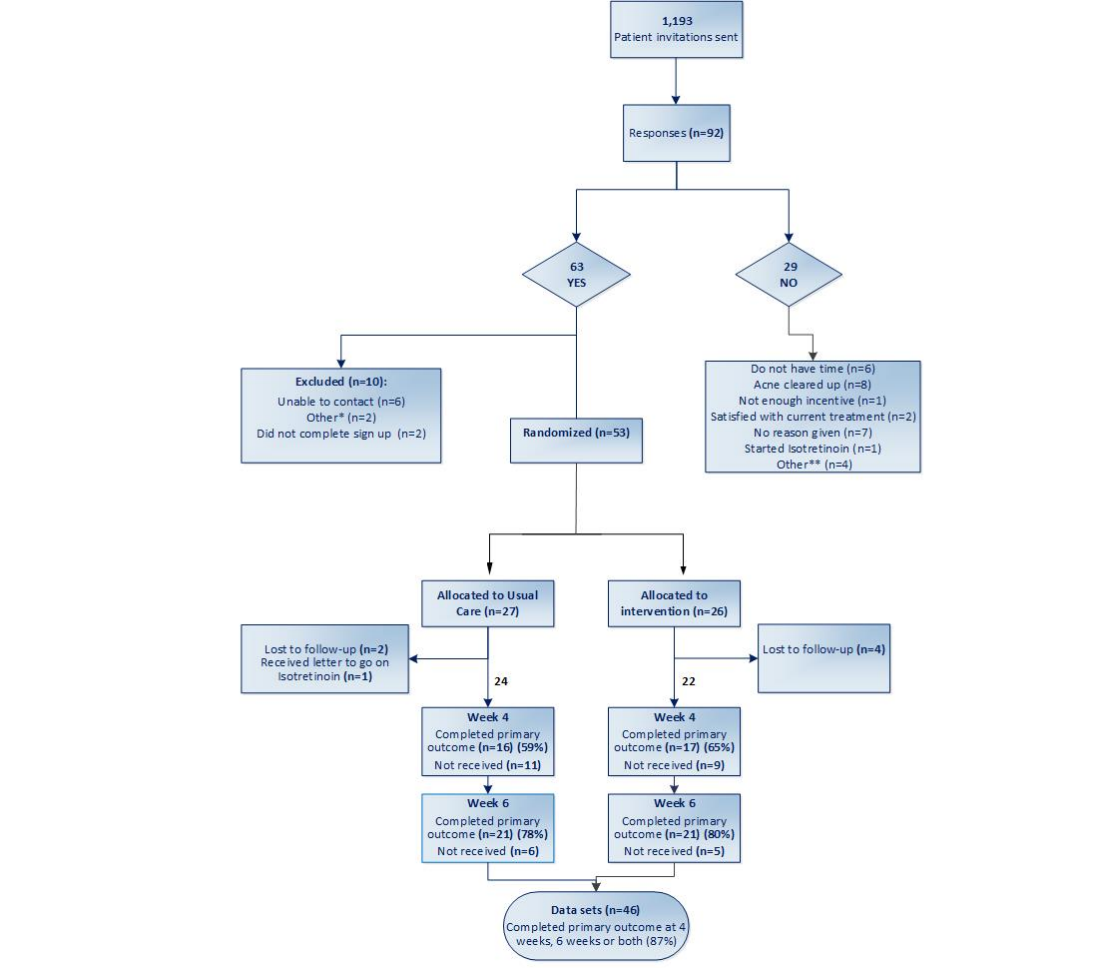
Flow diagram of recruitment process. *Problem with LifeGuide randomization procedure incurred delay and participants did not log back in; **Felt like homework; not planning on using topicals; not interested.

Recruitment took place from September 2018 to April 2019, and the follow-up ended in June 2019. In total, 1193 invitation letters were sent from 20 primary care practices in the South of England. Of the 1193 invitations sent, we received 92 (7.71%) responses, with 63 (5.28%) agreeing to take part and 29 (2.43%) giving reasons why they could not. Of the 63 participants, 53 (84%) registered on the web and were randomized (usual care: 27/53, 51%; usual care plus web-based intervention: 26/53, 49%). Of the 53 registered participants, 46 (87%) participants completed follow-up at 4 weeks, 6 weeks, or both time points (Figure 2). Five practices carried mail-out using the amended documents, which led to a small increase in participants signing up for the study—from 4.5% to 4.8%.

### Participant Characteristics

The sample comprised 72% (38/53) female and 28% (15/53) male participants with a mean age of 19 (SD 2.6) years. The mean age at the onset of acne was reported as 14 (SD 2.1) years. Of the 53 participants, 39 (74%) reported living at home, and 44 (83%) were in full-time education (Table 3).

**Table 3:** Participant characteristics at baseline (N=53).

Participant characteristics			Intervention (n=26)		Usual care (n=27)		Total (n=53)
**Gender, n (%)**							
	Female	21 (81)		17 (63)		38 (72)	
	Male	5 (19)		10 (37)		15 (28)	
Age (years), mean (SD)			18.3 (2.6)		18.8 (3.4)		18.6 (3)
Age at onset of acne (years), mean (SD)			13.54 (2.1)		13.8 (2.5)		13.7 (2.3)
**Living at home, n (%)**							
	Yes	21 (81)		18 (67)		39 (74)	
	No	5 (19)		9 (33)		14 (26)	
**Currently in full-time education, n (%)**							
	Yes	22 (85)		22 (82)		44 (83)	
	No	4 (15)		5 (19)		9 (17)	

### Questionnaire Completion

Baseline completion rates were high for all questionnaires (Table 4). Not all participants experienced side effects; therefore, the question about their management had a lower completion rate at each interval. Completion rates were higher at 6 weeks than at 4 weeks as there was a longer period to contact participants by phone to complete the questionnaires if they had not done so after receiving the reminder emails. At 4 weeks, 6% (1/17) of participants in the intervention group and 6% (1/16) of participants in the usual care group completed the primary outcome measure from the questionnaire over the phone. At 6 weeks, this was 24% (5/21) of participants in the intervention group and 14% (3/21) in the usual care group.

**Table 4:** Questionnaire completion rates (N=53).

Outcome measure	Baseline, n (%)	4 weeks, n (%)	6 weeks, n (%)
Overall Skindex-16	53 (100)	33 (62)	42 (79)
EQ-5D-5L	53 (100)	32 (60)	39 (74)
EQ VAS^a^	53 (100)	31 (59)	34 (64)
PHQ-4^b^	53 (100)	31 (59)	36 (68)
Credibility	53 (100)	N/A^c^	N/A
Expectancy	53 (100)	N/A	N/A
PETS^d^ symptoms (n=26)	26 (100)	17 (65)	17 (65)
PETS uncertainty (n=26)	25 (96)	17 (65)	17 (65)
PETS doubts (n=26)	25 (92)	17 (65)	17 (65)
PETS practical problems (n=26)	25 (96)	17 (65)	17 (65)
What topical using	53 (100)	31 (59)	36 (68)
How often using treatment	53 (100)	31 (59)	34 (64)
Side effects	51 (96)	31 (59)	32 (60)
Management of side effects (people who reported side effects)	31 (59)	19 (36)	25 (47)
Other treatment	53 (100)	30 (57)	37 (70)

^a^EQ VAS: EuroQol Visual Analogue Scale.

^b^PHQ-4: Patient Health Questionnaire-4.

^c^N/A: not applicable.

^d^PETS: Problematic Experiences Therapy Scale.

### Outcome Measures

#### Skindex-16

The Skindex-16 overall mean score at baseline was 55.4 (SD 21.8) across both groups. There was a substantial improvement in both groups, and the mean differences between groups, when controlling for baseline scores and covariates (gender, age, age onset, and education), suggested a trend toward benefit at both 4 and 6 weeks: at 4 weeks, the intervention group had a score 5.2 points lower (95% CI −14.58 to 4.09) than the usual care group and at 6 weeks 2.9 points lower (95% CI −13.27 to 7.47; Table 5).

**Table 5:** Scores at baseline and follow-up and estimate of mean differences controlling for baseline and covariates (n=53).

Score description			Baseline					4-week follow-up					4-week follow-up, controlling for baseline and other covariates, mean difference (95% CI)			6-week follow-up					6-week follow-up, controlling for baseline and other covariates, mean difference (95% CI)
			n value		Value, mean (SD)		n value			Value, mean (SD)					n value			Value, mean (SD)			
**Overall Skindex-16 scores**																					
	Usual care	27		55.4 (24)		16			54.2 (18.7)		N/A^a^			21			48 (23.8)		N/A		
	Web-based intervention	26		55.3 (19.8)		17			45.8 (19.9)		−5.2 (−14.58 to 4.09)			21			43.4 (22.2)		−2.9 (−13.27 to 7.47)		
**Skindex-16 symptom**																					
	Usual care	N/A		41.3 (25.5)		N/A			35.5 (21.5)		N/A			N/A			37.3 (24.3)		N/A		
	Web-based intervention	N/A		31.9 (19.8)		N/A			30.6 (24.1)		5.4 (−8.41 to 19.22)			N/A			27 (21.5)		−0.9 (−11.76 to 10.03)		
**Skindex-16 emotional**																					
	Usual care	N/A		72.7 (27.5)		N/A			74.2 (23.4)		N/A			N/A			63.6 (28.1)		N/A		
	Web-based intervention	N/A		76.6 (21.1)		N/A			63.7 (22.3)		−12.4 (−24.23 to −0.67)			N/A			62 (24.3)		−3.9 (−16.65 to 8.75)		
**Skindex-16 functioning**																					
	Usual care	N/A		42.6 (28.3)		N/A			41.2 (22.7)		N/A			N/A			34.8 (27.8)		N/A		
	Web-based intervention	N/A		44.1 (27.9)		N/A			31.9 (26.8)		−6.4 (−20.52 to 7.79)			N/A			30.5 (28.9)		−3.4 (−16.75 to 9.9)		
**PHQ-4^b^ total**																					
	Usual care	27		4 (3.5)		16			3.9 (3.3)		N/A			18			3.7 (3.3)		N/A		
	Web-based intervention	26		4.6 (3.7)		15			2.3 (2.9)		−1.7 (−3.66 to 0.18)			18			3.2 (3.3)		−0.8 (−2.6 to 0.97)		
**PETS^c^ symptoms**																					
	Usual care	26		3.9 (1)		14			4 (1.1)		N/A			17			4.1 (0.9)		N/A		
	Web-based intervention	26		3.9 (0.9)		17			4.2 (1.2)		0.2 (−0.65 to 1.15)			17			4.2 (0.9)		0.2 (−0.47 to 0.82)		
**PETS uncertainty**																					
	Usual care	26		4.5 (0.9)		15			4.5 (1.2)		N/A			18			4.2 (1.1)		N/A		
	Web-based intervention	25		4.4 (1)		17			4.7 (0.6)		0.1 (−0.51 to 0.67)			17			4.9 (0.2)		0.6 (0.19 to 1.08)		
**PETS doubt**																					
	Usual care	27		3.8 (1)		19			3.7 (1.1)		N/A			18			3.7 (1.1)		N/A		
	Web-based intervention	24		3.4 (1.3)		17			4.2 (0.8)		0.5 (−0.23 to 1.25)			17			4.2 (1)		0.5 (−0.18 to 1.24)		
**PETS practical problems**																					
	Usual care	27		3.4 (1.3)		15			3.6 (1.3)		N/A			18			3. (1.3)		N/A		
	Web-based intervention	25		3.8 (1)		17			4 (1.1)		0.1 (−0.44 to 0.73)			17			4.1 (1.1)		0.7 (0.02 to 1.3)		

^a^N/A: not applicable.

^b^PHQ-4: Patient Health Questionnaire-4.

^c^PETS: Problematic Experiences Therapy Scale.

#### Individual Subscales for Skindex-16

There was no evidence of a trend toward benefit in the symptoms subscale (intervention group 5.4 points higher at 4 weeks: 95% CI −8.41 to 19.22; 0.9 points lower at 6 weeks: 95% CI −11.76 to −10.03); however, some evidence of a trend toward benefit in the emotional subscale (intervention 12.4 points lower at 4 weeks: 95% CI −24.23 to −0.67; 3.9 points lower at 6 weeks: 95% CI −16.65 to 8.75) and functioning subscale (intervention group 6.4 points lower at 4 weeks: 95% CI −20.52 to 7.79; 3.4 points lower at 6 weeks: 95% CI −16.75 to 9.9; Table 5).

### Other Outcome Measures

The baseline mean score for anxiety and depression (PHQ-4) suggests that the overall scores between groups were in the mild range for anxiety and depression with a score of 4.3 (SD 3.6) and a trend toward improvement in the intervention group at 4 weeks compared with the usual care group. For all PETS subscales (symptoms, uncertainty, doubt, and practical problems), there were also suggestions of a trend toward benefit (Table 5).

### Treatment Monitoring

#### Topical Treatment Used

More people in the usual care group reported using topicals at baseline compared with those in the intervention group. In the intervention group, the percentage of people using topicals increased from baseline to 4 weeks by 13.5% and decreased by 0.8% in the usual care group (Table 6).

**Table 6:** Reported topical treatment use between groups at each interval.

Topical used			Intervention				Usual care		
			n (%)		N		n (%)		N
**Topical treatments**									
	Baseline	16 (62)		26		20 (74)		27	
	4 weeks	12 (75)		16		11 (73)		15	
	6 weeks	15 (88)		17		15 (79)		19	
**None**									
	Baseline	3 (12)		26		6 (22)		27	
	4 weeks	3 (19)		16		4 (27)		15	
	6 weeks	2 (12)		17		4 (21)		19	
**Other^a^**									
	Baseline	7 (27)		26		1 (4)		27	
	4 weeks	1 (6)		16		0 (0)		15	
	6 weeks	0 (0)		17		0 (0)		19	

^a^Other topical treatments including branded products.

#### Topical Treatment Side Effects and Management

At 4 and 6 weeks, the usual care groups reported similar rates of side effects compared with the intervention group (Table 7). There was an increase of 13.5% from baseline to 4 weeks in the number of people reporting continuing treatment (including altering application as advised by the website) when experiencing minor side effects compared with the usual, which decreased by 2.4% at 4 weeks (Table 8). In both groups, the most common frequency of application at all intervals was *once or more than once a day or most days*. The intervention group and the usual care decreased similarly in the number of people reporting application of *once or more than once a day or most days* at 4 weeks (Table 9).

**Table 7:** Reported side effects from topical treatments.

Side effects			Intervention					Usual care		
			n (%)		N		n (%)			N
**Topical treatments**										
	Baseline	15 (60)		25		16 (62)			26	
	4 weeks	9 (53)		17		8 (57)			14	
	6 weeks	10 (67)		15		12 (71)			17	
**None**										
	Baseline	10 (40)		25		10 (39)			26	
	4 weeks	8 (47)		17		6 (43)			14	
	6 weeks	5 (33)		15		5 (29)			17	

**Table 8:** Reported management of side effects from topicals.

Management of side effects			Intervention					Usual care		
			n (%)		N		n (%)			N
**Continued treatment**										
	Baseline	9 (64)		14		14 (82)			17	
	4 weeks	7 (78)		9		8 (80)			10	
	6 weeks	7 (64)		11		10 (71)			14	
**Stopped treatment**										
	Baseline	5 (36)		14		2 (12)			17	
	4 weeks	2 (22)		9		0 (0)			10	
	6 weeks	2 (18)		11		1 (7)			14	
**Other^a^**										
	Baseline	2 (14)		14		1 (6)			17	
	4 weeks	1 (11)		9		2 (20)			10	
	6 weeks	1 (9)		11		3 (21)			14	

**^a^**Other management included using moisturizer, hydrating masks, or face washes.

**Table 9:** Reported frequency of application of topicals.

Frequency of application		Intervention			Usual care	
		n (%)	N	n (%)		N
**Once or more than once a day or most days**						
	Baseline	19 (73)	26	19 (70)		27
	4 weeks	11 (65)	17	9 (64)		14
	6 weeks	13 (81)	16	12 (67)		18
**Not at all or once or twice a week**						
	Baseline	7 (27)	26	8 (30)		27
	4 weeks	6 (35)	17	5 (36)		14
	6 weeks	3 (19)	16	6 (33)		18

### Intervention Use

Approximately 88% (23/26) of participants in the intervention group completed the core module *core treatments*. Completion was decided based on whether participants clicked through to the end of the core module pages without logging off the web-based intervention. Approximately 69% (18/26) of participants visited the website three times or more, including baseline visits. There was a low uptake of the 4-week challenge (38%), although this was based on whether participants entered a start date; however, it is possible that some participants engaged without entering a start date. Visits to some of the optional modules were low: 42% of participants accessed the module on *living with spots or acne,* and more than a quarter viewed the *myth-busting quiz*; fewer were interested in *talking to your GP* (Table 10).

**Table 10:** Intervention use (N=26).

Measures of intervention use			Web-based intervention, n (%)
Core module completed			23 (88)
**Total number of visits to intervention**			
	1	3 (12)	
	2	5 (19)	
	3	7 (27)	
	4	7 (27)	
	5	2 (8)	
	6	2 (8)	
Signed up to 4-week challenge			10 (38)
**Visits to other modules**			
	Living with spots or acne	11 (42)	
	Myth-busting quiz	7 (27)	
	What are spots or acne	7 (27)	
	Other treatments	7 (27)	
	Oral antibiotics	5 (19)	
	Talking to your GP^a^	3 (12)	

^a^GP: general practitioner.

## Discussion

### Principal Findings

To our knowledge, this is the first web-based behavioral intervention developed for young people with acne, using the PBA along with theory and evidence [22]. The recruitment rate of 8% was lower than expected; however, retention rates for people completing the primary outcome measure at either 4 or 6 weeks were high (87%). There was a suggestive trend toward benefit in the primary (Skindex-16) and secondary outcome measures (PHQ-4 and PETS) when looking at the mean differences. More people in the intervention group reported using topical treatments, and they were also more likely to manage side effects from topical treatments by continuing treatment as opposed to stopping treatment compared with the usual care group. Completion of the core module was high (88%), although it was low for the optional modules. Although promising, these findings should be viewed with caution, as this study was not powered to determine effectiveness.

### Limitations

There were several limitations and changes that should be considered based on the findings of this feasibility trial. First, the mail-out through primary care practices received a low response rate, suggesting that people who took part in the trial may be more motivated and possibly have higher literacy than those who did not respond. Therefore, the sample may not be fully representative of young people who consult primary care for their acne. A key reason for not participating was time commitment, which suggests that the level of involvement in the study may need to be made clearer. Another reason for not participating was that some participants’ skin had cleared up. This could be a reflection on the search strategy or the unpredictable nature of their skin condition. The changes to the recruitment process led to a slight increase in response rate which suggests that if implemented earlier this could have potentially improved the numbers recruited. People who took part in the study also seemed to be using topical treatments already, which suggests that recruitment in a future trial should seek participants who are not already using them to benefit from the intervention. We may also need to consider other ways of reaching the target population, including other platforms such as social media, pharmacies, and schools.

Second, there was a low uptake of the optional modules, which suggests that the intervention may need to be refined further. However, the reason for including these modules as optional was that they might not be applicable to everyone at that time but were seen as important in earlier qualitative research. Uptake of the *4-week challenge* was low; however, this was only determined by people entering a date to start the challenge. In the future, this should be monitored more closely, and perhaps there should be a question in the survey to identify those who did and did not take part. It is also unclear whether people in the usual care group attended their GP practices and were prescribed treatment as usual, making it difficult to fully understand why people in the usual care increased on a number of outcome measures.

Although the target sample size was not reached, this was a feasibility study and provided useful information about the changes that need to be considered for a future trial. Owing to the randomization error, participants were randomized in a 1:1 ratio instead of 2:1 for intervention to usual care group. This resulted in less usage data for the intervention group, which could have provided further information on intervention use. Although there was a trend toward benefit in both the primary and secondary outcome measures, a larger sample is needed to draw conclusions about the effect of the intervention.

### Comparison with Prior Work

The findings from this feasibility trial reflect the results of previous trials testing the effectiveness of interventions for acne [11,13-15,19]. For example, a pilot RCT of an interactive health education tool also found that those in the intervention group had improved QoL scores compared with the control group, although these findings were not statistically significant [11]. However, this study did not specify which treatments were being used by participants in the intervention (topical or oral treatments); therefore, comparisons should be made with caution. In this study, we used PETS scores to determine adherence to topical treatments, which suggested a trend in the direction of benefit. A previous RCT investigating the effectiveness of supplementary educational materials on a combination topical treatment also found improved adherence, although using an objective measure (medication event monitoring system) [10]. There is currently no standardized or fully validated method of measurement for adherence to acne treatments [46], and further work would benefit in addressing this so that heterogeneity and adherence can be compared across trials.

The rate of follow-up in this study was high at 6 weeks (79%) in terms of those completing the primary outcome measure (Skindex-16). This is in line with a previous trial that found a follow-up rate of 84.5% when recruiting through primary care [10]. Similarly, a study investigating adherence rates using an internet-based survey for young people with acne had a follow-up rate of 75%, although it is unclear where participants were recruited from, and the sample size was small with 20 participants [14].

### Conclusions

This feasibility trial demonstrates that a web-based behavioral intervention for young people with acne can be delivered with high retention, high engagement with the core module, and trends in the direction of benefit for the primary outcome measure. However, recruitment to this study was challenging, and alternative methods of seeking participants should be considered for a full-scale trial of a similar intervention, particularly when seeking a population less likely to be using effective topical treatments for acne.

## Appendix

Multimedia Appendix 1 CONSORT-EHEALTH checklist (V 1.6.1).

## Abbreviations

GP: general practitioner
PBA: Person-based approach
PETS: Problematic Experiences Therapy Scale
PHQ-4: Patient Health Questionnaire-4
PROSPERO: International Prospective Register of Systematic Reviews
QoL: quality of life
RCT: randomized controlled trial
TIDieR: Template for Intervention Description and Replication
